# Effect of Vitamin D Supplementation on Falls, Physical Function, and Activity: Results From the STURDY Trial

**DOI:** 10.1093/geroni/igaa057.2736

**Published:** 2020-12-16

**Authors:** Jennifer Schrack, Lawrence Appel, Lewis Lipsitz

## Abstract

Each year, 2.8 million older adults are treated for falls, with over 800,000 hospitalized. Evidence suggests vitamin D supplementation might reduce the risk of falls, potentially through improvements in skeletal muscle function; however, results are inconsistent. In 2013 the NIA issued a request for applications to assess the efficacy and dose-response of vitamin D supplementation for fall prevention across a range of doses and serum 25(OH)D concentrations, resulting in the funding of STURDY (Study To Understand Fall Reduction and Vitamin D in You). STURDY was a seamless dose-finding and confirmatory, double-masked, response adaptive Bayesian randomized trial designed to find the best dose of vitamin D supplementation for fall prevention. Participants (n=688, ≥70 years with serum 25(OH)D of 10-29 ng/mL) were randomized to 200 (control), 1000 , 2000, or 4000 IU/day of vitamin D3.The first participant was randomized on 10/30/2015 and data collection ended on 5/31/2019. The primary outcome was time to first fall or death, and the secondary outcome was gait speed. Dr. Appel will present the main findings of the effect of vitamin D supplementation on time to first fall. Dr. Wanigatunga will present a more detailed analysis of the effect of vitamin D supplementation on fall characteristics, including indoor vs. outdoor falls, consequential falls, and repeat fall risk. Dr. Guralnik will present the effect of vitamin D supplementation on physical functioning, including gait speed, SPPB, 6-minute walk, and TUG performance. Dr. Schrack will present the effect of vitamin D supplementation on objectively measured physical activity.

